# Supplementary material for: Primary and Recall Immune Responses to SARS-CoV-2 in Breakthrough Infection
Source: Vaccines (Basel). 2023 Nov 9;11(11):1705. doi: 10.3390/vaccines11111705 (PMC10675240; doi:10.3390/vaccines11111705)
Supplement: Supplementary file 1 [file vaccines-11-01705-s001.zip › vaccines-2673961-supplementary.pdf]

Supplementary Materials

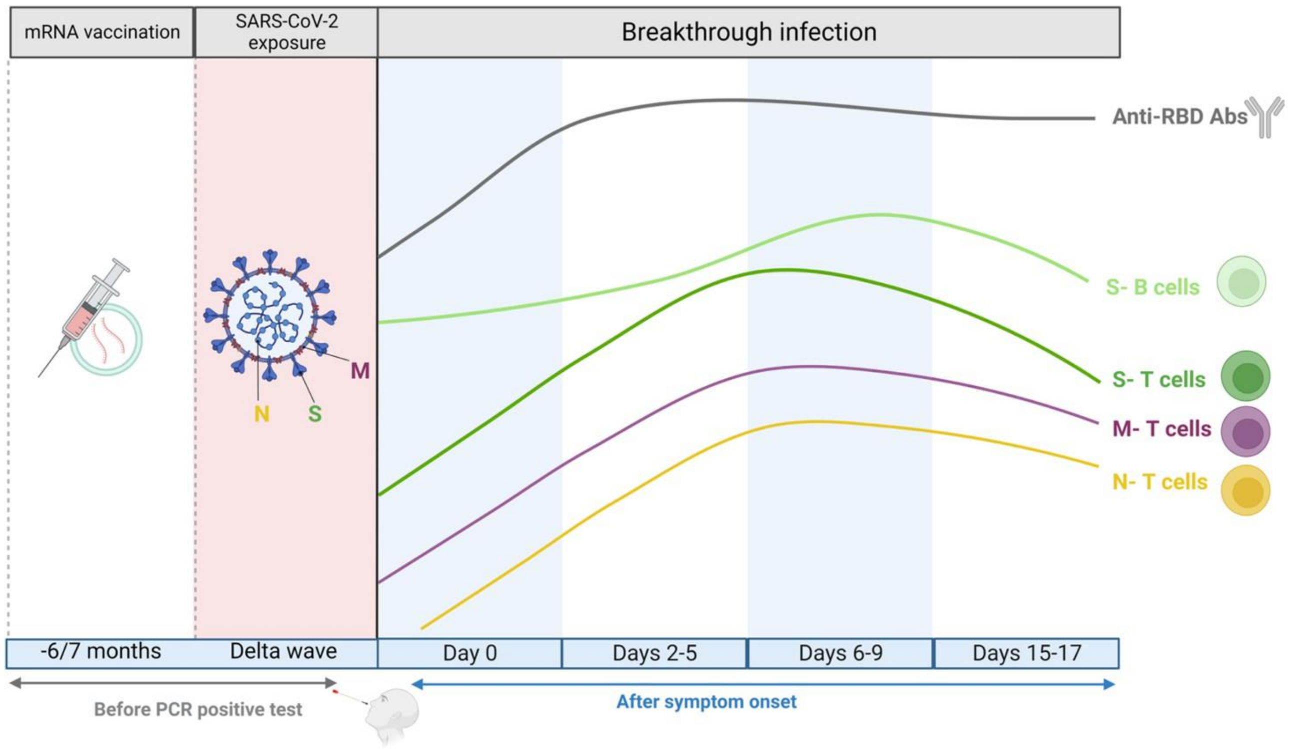

Figure S1. Study Design. Fifteen health care workers previously vaccinated with 2 doses of mRNA vaccine and with breakthrough infections, were followed longitudinally from day 0 (first positive PCR-based test) to negativization. The kinetics of the humoral and cellular response were assessed by grouping the longitudinal samples in four different time frames: 0, 2–5, 6-9 and 15–17 days. (S-: Spike, M-: Membrane, N-:Nucleocapsid, RBD-: Receptor Binding Domain).

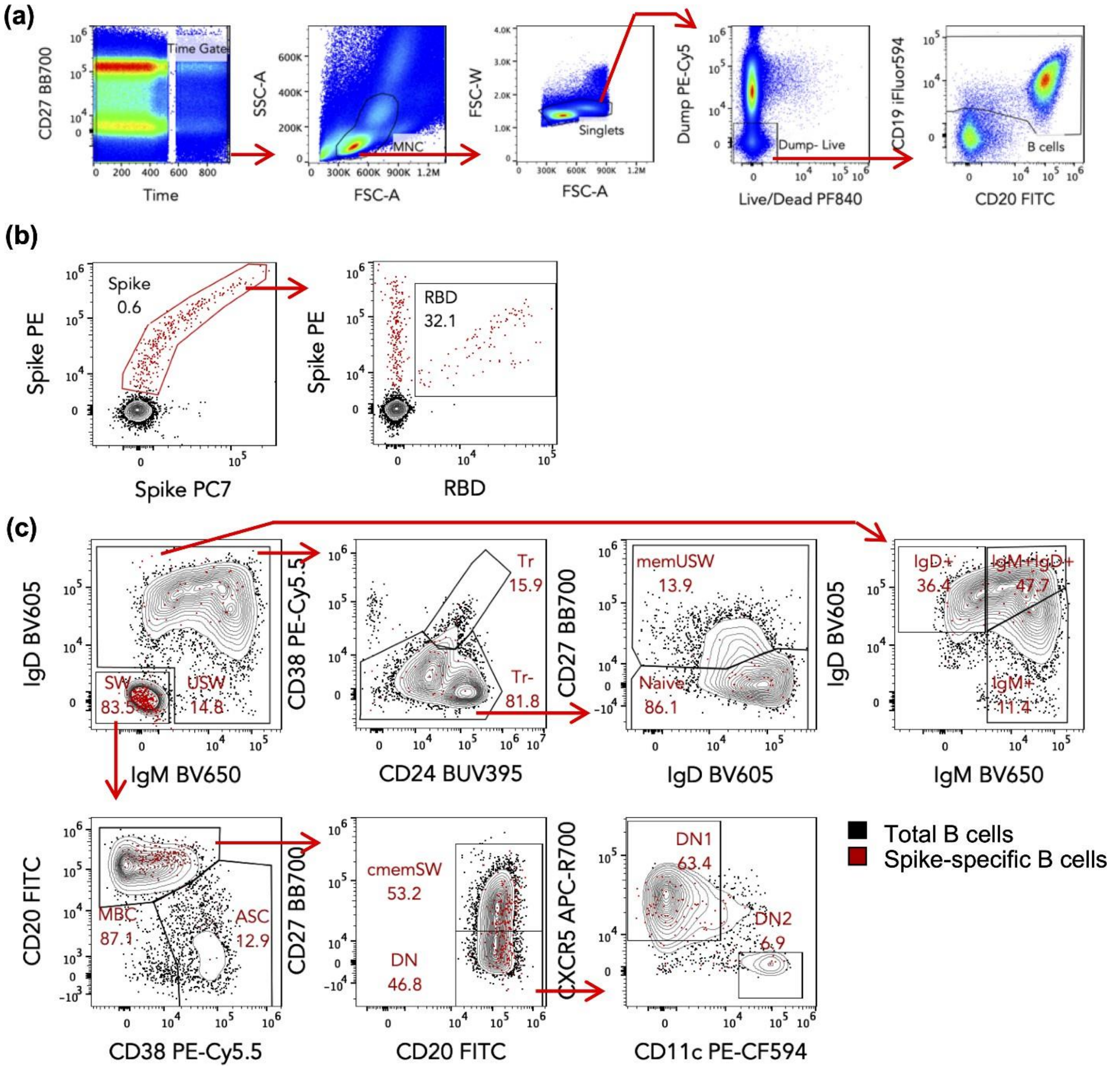

Figure S2. Gating strategy of antigen-specific B cell subpopulations**. (a)** Gating strategy for the identification of CD20+ CD19+ B cells on viable (Live/Dead) and single lymphocytes. Monocytes, neutrophils, platelets, red blood and T cells are removed by exclusion of CD14+, CD16+, CD41+, CD235a+ and CD3+ cells. **(b)** Gating of S- and RBD-specific B cells. **(c)** Sequential gating and characterization of S-specific B cells (red dots), overlaid on total CD19+ B cells (grey contour plots with outliers). Red numbers in plots under subset names indicate the frequency of S-specific B cells. (RBD: Receptor Binding Domain, ASC: Antibody Secreting Cells, cmemSW: classical memory switched, DN1: double negative 1, DN2: double negative 2, Tr: transitional, USWmem: unswitched memory).

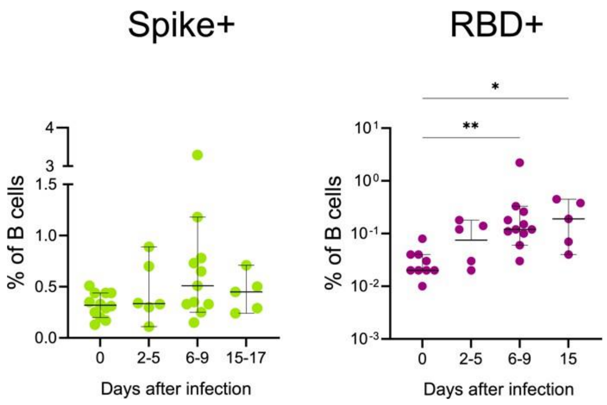

Figure S3. Frequencies of Spike and RBD-specific B cells. Values were compared by non parametric repeated measures Kruskall-Wallis and corrected for Dunn's multiple comparison tests; * $p < 0.05$; ** $p < 0.01$. (RBD: Receptor Binding Domain).

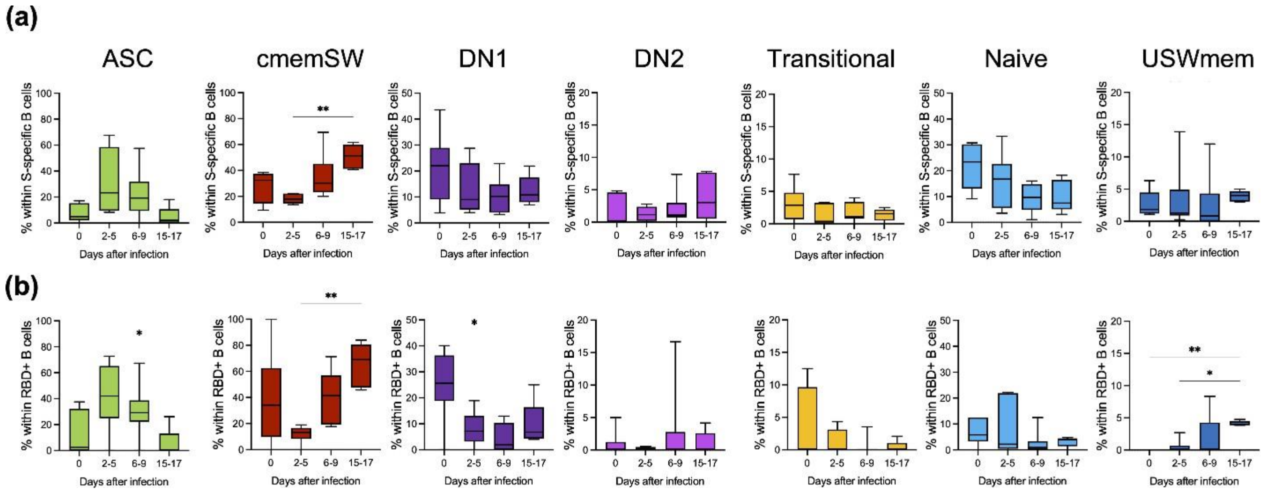

Figure S4. Frequency of different B cell subpopulations. **(a)** Frequency of different B cell subpopulations within S-specific B cells (day 0 *n* = 6; 2–5 days *n* = 6; 6–9 days *n* = 11; 15–17 *n* = 5). Values were compared by nonparametric repeated measures Kruskall-Wallis and corrected for Dunn's multiple comparison tests; ** $p < 0.005$; **(b)** Frequency of different B cell subpopulations within RBD-specific B cells (day 0 *n* = 6; 2–5 days *n* = 6; 6–9 days *n* = 11; 15–17 *n* = 5). Values were compared by nonparametric repeated measures Kruskall-Wallis and corrected for Dunn's multiple comparison tests; * $p < 0.05$; ** $p < 0.01$. (RBD: Receptor Binding Domain, ASC: Antibody Secreting Cells, cmemSW: classical memory switched, DN1: double negative 1, DN2: double negative 2, Tr: transitional, USWmem: unswitched memory).

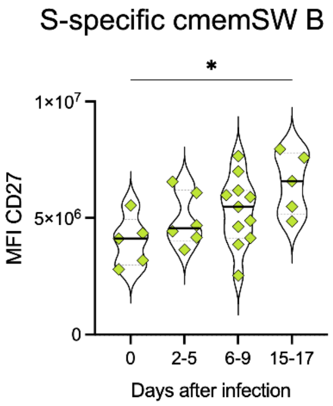

Figure S5. MFI of CD27. MFI of CD27 within S-cmemSW B cell subset; Values were compared by nonparametric repeated measures Kruskall-Wallis and corrected for Dunn's multiple comparison tests; * $p < 0.05$. (S-: Spike, cmemSW: classical memory switched).

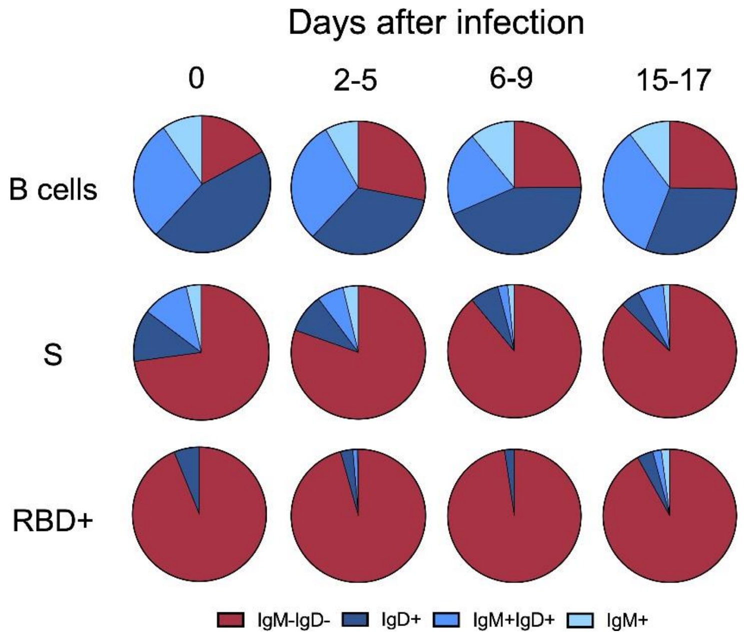

Figure S6. Frequency of different Ig isotype expressed on surface. Frequency of IgM-IgD-, IgD+ single positive, IgM+ single positive and IgM+IgD+ within total B cells, S- and RBD-specific B cells (day 0 n = 6; 2–5 days n = 6; 6–9 days n = 11; 15–17 n = 5). (S-: Spike, RBD-: Receptor Binding Domain).

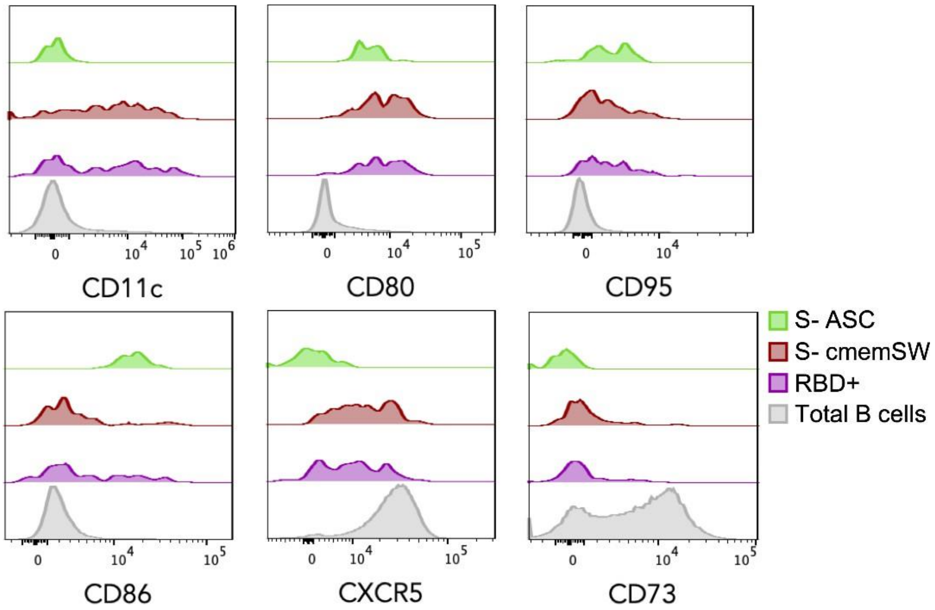

Figure S7. CD11c, CD80, CD95, CD86, CXCR5 and CD73 expression in S-specific subpopulations and in total B cells. Overlaid Median Fluorescence Intensity (MFI) of each marker is represented in S- specific subpopulations (S- ASCs and S- cmemSW), RBD+ and total B cells, identified by different colours as shown in the legend. (S-: Spike, RBD: Receptor Binding Domain, ASC: Antibody Secreting Cells, cmemSW: classical memory switched).

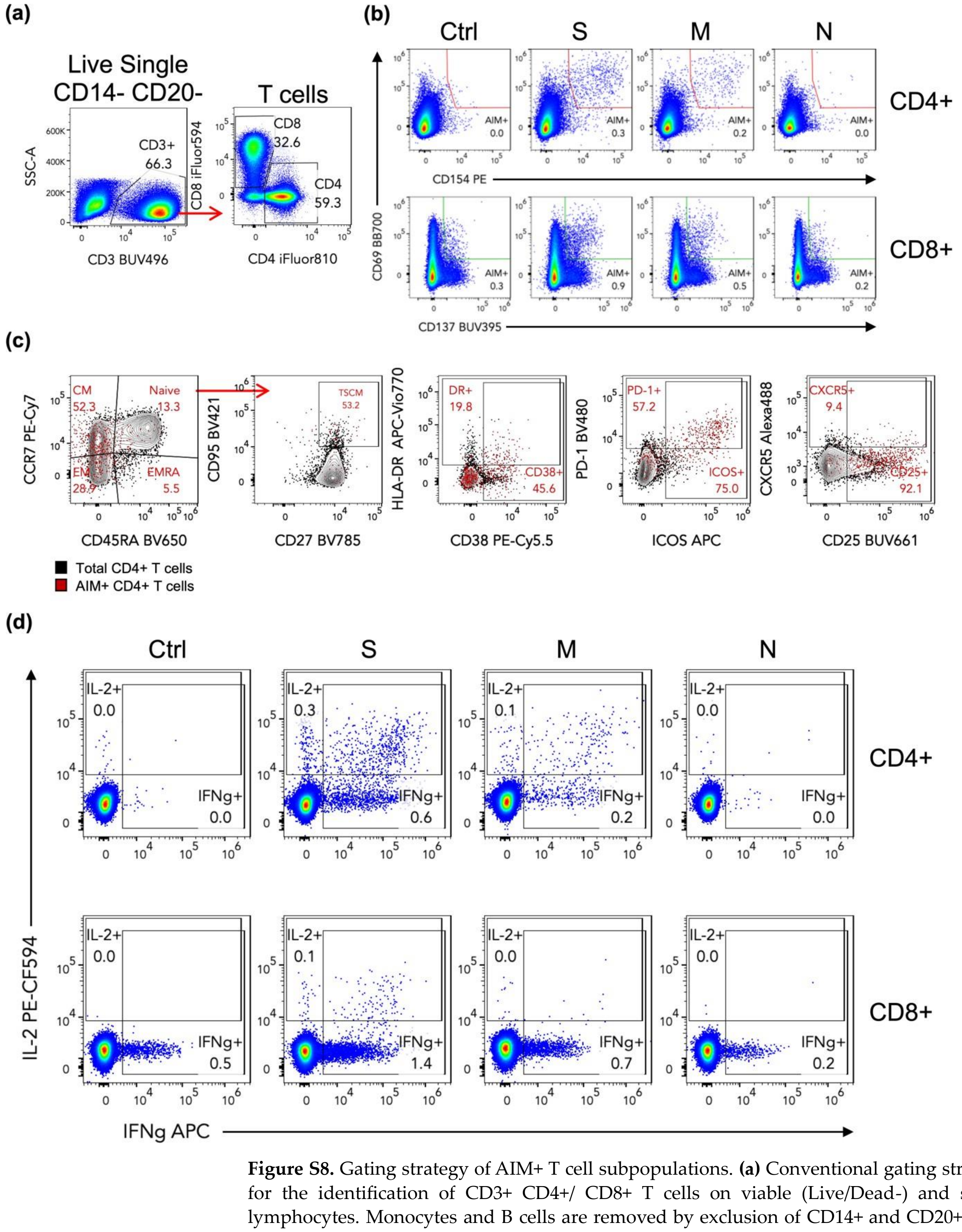

Figure S8. Gating strategy of AIM+ T cell subpopulations. **(a)** Conventional gating strategy for the identification of CD3+ CD4+/ CD8+ T cells on viable (Live/Dead-) and single lymphocytes. Monocytes and B cells are removed by exclusion of CD14+ and CD20+ cells. (**b**) Gating and characterization of AIM+ CD4+ and of AIM+ CD8+ T cells in unstimulated (Ctrl) and stimulated (S, M and N) conditions. **(c)** Representative gating and characterization of activation/homing markers (HLA-DR, CD38, PD-1, ICOS, CXCR5, CD25, CCR7 and CD45RA) in AIM+ CD4+ T cells. Red dots represent AIM+ cells overlaid on total CD4+ cells (grey contour plots with outliers). Numbers inside or adjacent to the gates indicate frequencies within AIM+ T cells. **(d)** IFN-γ+ and IL-2+ production by both CD4+ and CD8+ T cells in unstimulated (Ctrl) and stimulated (S, M and N) conditions. Numbers inside or adjacent to the gates indicate frequencies within CD4+ or CD8+ T cells. (S-: Spike, M-: Membrane, N-:Nucleocapsid, AIM: Activation Induced Markers, TSCM: T Stem Cell Memory, EM: Effector Memory, CM: Central Memory, EMRA: terminally differentiated effector memory).

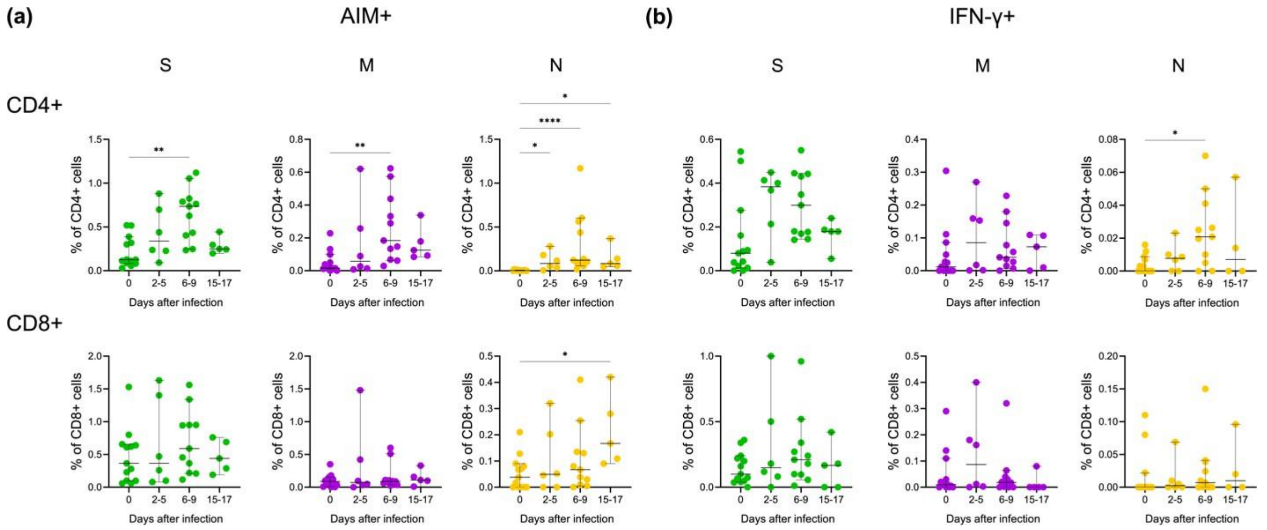

Figure S9. Frequencies of AIM+ T cells. **(a)** Frequencies of AIM+ CD4+ (top) or CD8+ (bottom) T cells specific for S, N, and M antigens. **(b)** Frequencies of IFN-γ+ CD4+ (top) or CD8+ (bottom) T cells specific for S, N, and M antigens. Values obtained at each time point for S-, M- and N-specific T cells were compared by non- parametric Kruskall-Wallis tests followed by Dunn's post hoc tests. * $p < 0.05$; **** $p < 0.0001$; no symbol, not significant. (S-: Spike, M-: Membrane, N-:Nucleocapsid, AIM: Activation Induced Markers)

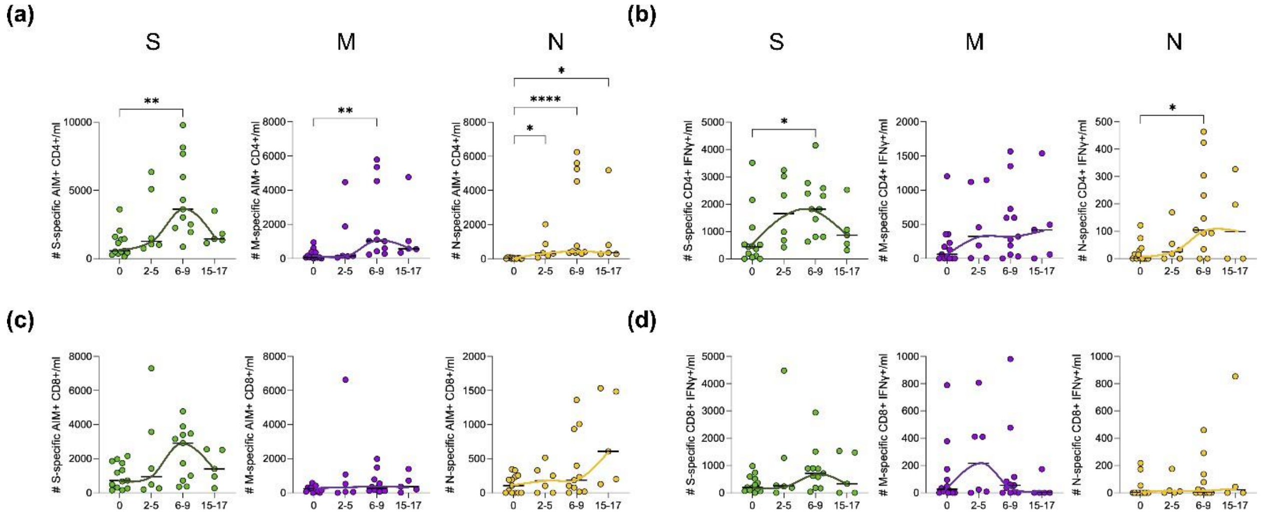

Figure S10. Kinetics of Spike-specific, membrane-specific and nucleocapsid-specific CD4+ and CD8+ T cells during breakthrough infection. Numbers of S-, M-, and N-specific CD4+ (A) and CD8+ The curves connect the medians of the observations over time and are for illustrative purposes only. The non-parametric Kruskal- Wallis test followed by Dunn's post hoc tests was used to compare time points. * $p < 0.05$; *** $p < 0.001$, **** $p < 0.0001$; no symbol, not significant. (S-: Spike, M-: Membrane, N-:Nucleocapsid, AIM: Activation Induced Markers).

Table S1. Donor characteristics.

Donor	Sex	Age
R_01	M	35
R_02	F	22
R_03	M	23
R_04	F	25
R_05	F	23
R_06	F	52
R_07	M	35
R_08	F	31
R_09	F	56
R_10	F	54
R_11	F	31
R_12	M	60
R_13	M	49
R_14	F	52
R_15	F	45

Table S2. Reagents for the detection and study of antigen-specific B cells.

Target	Fluorochrome	Dilution	Clone	Manufacturer
CD20	FITC	1:200	2H7	Bect.Dick.
CD11c	PE-CF594	1:250	B-LY6	Bect.Dick.
CD27	BB700	1:120	M-T271	Bect.Dick.
Streptavidin	PE	-	-	Miltenyi
CD19	iFluor594	1:30	HI19A	AAT Bioquest
CD3	PE-Cy5	1:100	UCHT1	Coulter
CD14		1:100	RMO52	Coulter
CD16		1:100	3G8	Bect.Dick.
CD41a		1:200	HIP8	Bect.Dick.
CD235a		1:500	GA-R2 (HIR2)	Bect.Dick.
CD38	PE-Cy5.5	1:100	LS198-4-3	Coulter
Streptavidin	PE-Vio770	-	-	Miltenyi
CD73	APC	1:100	AD2	Miltenyi
CXCR5	APC-R700	1:60	RF8B2	Bect.Dick.
CD80	BV421	1:30	L307.4	Bect.Dick.
IgD	BV605	1:50	IA6-2	Bect.Dick.
IgM	BV650	1:120	G20-127	Bect.Dick.
CD95	BV786	1:30	DX2	Bect.Dick.
CD24	BUV395	1:60	ML5	Bect.Dick.
CD86	BUV496	1:180	2331 (FUN-1)	Bect.Dick.
Streptavidin	iFluor810	-	-	AAT Bioquest
Live Dead	Promo Fluor 840	1:10,000	NA	Promokine

Table S3. Reagents for AIM assay.

Target	Fluorochrome	Dilution	Clone	Manufacturer
CD3	BUV496	1:50	UCHT1	Bect.Dick.
CD4	iFluor810	1:50	RPA-T4	AAT Bioquest
CD8	iFluor594	1:150	SK1	AAT Bioquest
CD14/CD20	PE-Cy5	1:50	RM052/B9E9	Coulter
CD25	BUV661	1:30	2A3	Bect. Dick.
CD27	BV785	1:30	0323	Bect. Dick.
CD38	PE-Cy5.5	1:100	LS198-4-3	Coulter
CD45RA	BV650	1:200	HI100	Bect. Dick.
CD69	BB700	1:100	FN50	Bect. Dick.
CD95	BV421	1:30	DX2	Sony
CD137	BUV395	1:30	4B4-1	Bect. Dick.
CD154	PE	1:30	24-31	eBioscience
CXCR5	Alexa488	1:50	RF8B2	Bect. Dick.
CCR7	PE-Cy7	1:30	G043H7	SONY
ICOS	APC	1:100	ISA-3	Coulter
PD-1	BV480	1:30	EH12.1	Bect. Dick.
HLA-DR	APC-Vio770	1:120	REA805	Miltenyi
Live Dead	Promo Fluor 840	1:10,000	NA	Promokine

Table S4. Reagents for Absolute counts.

Target	Fluorochrome	Dilution	Clone	Manufacturer
CD3	APC-Vio770	1:120	REA613	Miltenyi
CD4	BV650	1:100	SK3	Bect.Dick.
CD8	APC	1:100	BW135/80	Miltenyi
CD19	PE-Cy7	1:100	J3-119	Coulter
CD45	FITC	1:50	KC56	Coulter

Table S5. Reagents for intracellular staining.

Target	Fluorochrome	Dilution	Clone	Manufacturer
CD3	BUV496	1:50	UCHT1	Bect.Dick.
CD4	iFluor810	1:50	RPA-T4	AAT Bioquest
CD8	IFluor594	1:150	SK1	AAT Bioquest
CD14	BUV395	1:50	MϕP9	Bect.Dick.
IL-2	PE-CF594	1:50	5344.11	Bect.Dick.
IFN-γ	APC	1:100	B27	Bect.Dick.
Live Dead	Promo Fluor 840	1:10,000	NA	Promokine
